# Impact of an mHealth Platform for Pregnancy on Nutrition and Lifestyle of the Reproductive Population: A Survey

**DOI:** 10.2196/mhealth.5197

**Published:** 2016-05-27

**Authors:** Matthijs R Van Dijk, Nicole A Huijgen, Sten P Willemsen, Joop SE Laven, Eric AP Steegers, Régine PM Steegers-Theunissen

**Affiliations:** ^1^ Erasmus Medical Center (MC) Department of Obstetrics and Gynecology University Medical Center Rotterdam Netherlands; ^2^ Erasmus Medical Center (MC) Biostatistics University Medical Center Rotterdam Netherlands

**Keywords:** preconception care, nutrition, lifestyle, mHealth, pregnancy

## Abstract

**Background:**

Poor nutrition and lifestyle behaviors exert detrimental effects on reproduction and health during the life course. Therefore, lifestyle interventions during the periconceptional period can improve fertility, pregnancy outcome, and health of subsequent generations.

**Objective:**

This survey investigates the compliance, usability, and initial effectiveness of the Web-based mHealth platform, Smarter Pregnancy.

**Methods:**

A free subscription to the mHealth platform, Smarter Pregnancy, was provided to couples contemplating pregnancy (n=1275) or already pregnant (n=603). After baseline identification of inadequate nutrition and lifestyle behaviors, a personal online coaching program of 6 months was generated. Using multiple imputation and the generalized estimating equation model with independent correlations, we estimated the changes from inadequate to adequate nutrition and lifestyle behaviors over time. Subgroup analyses were performed for (1) overweight and obese women (body mass index [BMI] ≥25 kg/m^2^), (2) pregnant women at the start of the program, and (3) couples.

**Results:**

A 64.86% (1218/1878) compliance rate was observed and 54.7% (range 39.2-73.4%) of participants rated the program usability as positive or very positive. Adequate nutrition and lifestyle behaviors at baseline were 21.57% (405/1878) for vegetable intake, 52.61% (988/1878) for fruit intake, 85.44% (1303/1525) for folic acid use, 86.79% (1630/1878) for no tobacco use, and 64.43% (1210/1878) for no alcohol consumption. After 6 months of coaching, these lifestyle behaviors improved by 26.3% (95% CI 23.0-29.9) for vegetable intake, 38.4% (95% CI 34.5-42.5) for fruit intake, 56.3% (95% CI 48.8-63.6) for folic acid use, 35.1% (95% CI 29.1-41.6) for no tobacco use, and 41.9% (95% CI 35.2-48.9) for no alcohol consumption. The program showed the strongest effectiveness for participating couples.

**Conclusions:**

This novel Web-based mHealth platform shows high compliance and usability, and users demonstrate improvements in nutrition and lifestyle behaviors. The next step will be further validation in randomized controlled trials and implementation.

## Introduction

Worldwide, more than 45 million couples are contemplating pregnancy, of which around 22 million remain involuntarily childless. Moreover, of the more than 360 million pregnancies worldwide per year, at least 90 million end in miscarriage, 18 million result in congenital malformation, and 40 million result in children small for their gestational age. These reproductive and pregnancy failures largely originate in the periconceptional period, during which development and function of gametes, embryonic organs, and the placenta are programmed [1]. Poor periconceptional nutrition and lifestyle not only affect fertility and pregnancy outcome, but can also derange epigenetic programming with long-lasting health consequences [2]. Therefore, effective nutrition and lifestyle interventions in particular during this window of time will be an investment in healthy pregnancy and the health of current and future generations.

Currently, the most effective preconceptional interventions comprise weight loss, improvement of nutrition, use of folic acid supplements, and lowering the use of tobacco [3,4]. Unfortunately, women and men contemplating pregnancy or pregnant couples, as well as health care professionals, are often not aware of the detrimental effects of poor lifestyle behaviors [5-7]. These behaviors often accumulate not only in an individual, but also in couples, in particular among those with a low socioeconomic status, increasing the risk of a poor pregnancy outcome [8,9]. Therefore, it should be the responsibility of both health care professionals and patients to improve inadequate nutrition and lifestyle. To this aim, we previously developed and implemented a specific preconception outpatient clinic tailored to improve nutrition and lifestyle, which showed a 30% reduction of inadequate nutrition and lifestyle and a 65% increased chance of ongoing pregnancy after in vitro fertilization (IVF) treatment [6,10]. Obstacles of lifestyle counseling as part of periconceptional (clinical) care, however, require special expertise and time without reimbursement of costs.

Mobile health (mHealth) has the potential to transform health care delivery and to overcome obstacles by providing individual, tailored, and repeated information. Evidence is accumulating that mobile technology can effectively improve inadequate nutrition, lifestyle, and medication adherence [11]. Therefore, we developed the online, device-independent, Web-based coaching platform, *Smarter Pregnancy* [12]. This platform was based on scientific evidence of effective nutrition and lifestyle interventions, prevention and educational programs for noncommunicable diseases [13,14], and behavioral models, as well as our experience from the preconception outpatient clinic [6,15]. This mHealth platform aims to empower women, men, and health care professionals to improve inadequate nutrition and lifestyle. It also demonstrates the need for easily accessible, evidence-based interventions to improve the quality and effectiveness of periconceptional (clinical) care, the success of reproduction and pregnancy outcomes, as well as the prevention of disease during the life course [16,17].

Here we investigate the compliance, usability, and initial effectiveness of the Dutch version of this Web-based mHealth platform on changing inadequate nutrition and lifestyle behaviors in prepregnant women and their partners.

## Methods

### Study Population

In 2012 and 2013, women and men contemplating pregnancy or pregnant couples living in Rotterdam, the Netherlands, visiting the Erasmus Medical Center (MC), University Medical Center, or midwifery practices in Rotterdam, were recruited to the study. Recruits were invited to sign up for a free subscription to the Web-based Smarter Pregnancy platform [12]. This included 6 months of coaching on the most prevalent inadequate nutrition and lifestyle behaviors (ie, vegetable, fruit, and alcohol intake) or the most strongly demonstrated associations of behaviors with fertility and pregnancy course and outcome (ie, tobacco and folic acid supplement use).

Adequate daily intakes are defined as at least 200 grams of vegetables and at least two pieces of fruit, a folic acid supplement of 400 µg, and no tobacco or alcohol use [18]. Men were screened on the same behaviors, except for folic acid supplement use. Evaluation of the results of the baseline survey and the four follow-up screening surveys are shown on each participant's personal page as lifestyle risk scores in graphs and text, accompanied by personal advice according to preconceptional recommendations and Dutch guidelines [18]. If a participant completes the final screening survey at 6 months, we consider this as maximum compliance. More details are described in the next paragraph.

### Smarter Pregnancy

The coaching model developed for the Smarter Pregnancy platform is based on our research and expertise from the last 25 years on the impact of nutrition and lifestyle on reproduction as well as on pregnancy course and outcome [6,10,15,19,20]. In addition, we incorporated the following into the platform: results from the literature, Prochaska and Diclemente’s transtheoretical model with a focus on the readiness for behavioral change, Bandura’s social cognitive theory for self-efficacy, and Fogg's behavior model to include triggers to motivate and increase the ability to change [21-23]. Features of the attitude, social influence, and self-efficacy (ASE) model for coaching are applied; the ASE model has been frequently used for developing health education and prevention. Elements of this model comprise individual attitude, social influence, and self-efficacy aimed at the understanding and motives of people to engage in specific behavior [24].

**Figure 1:**
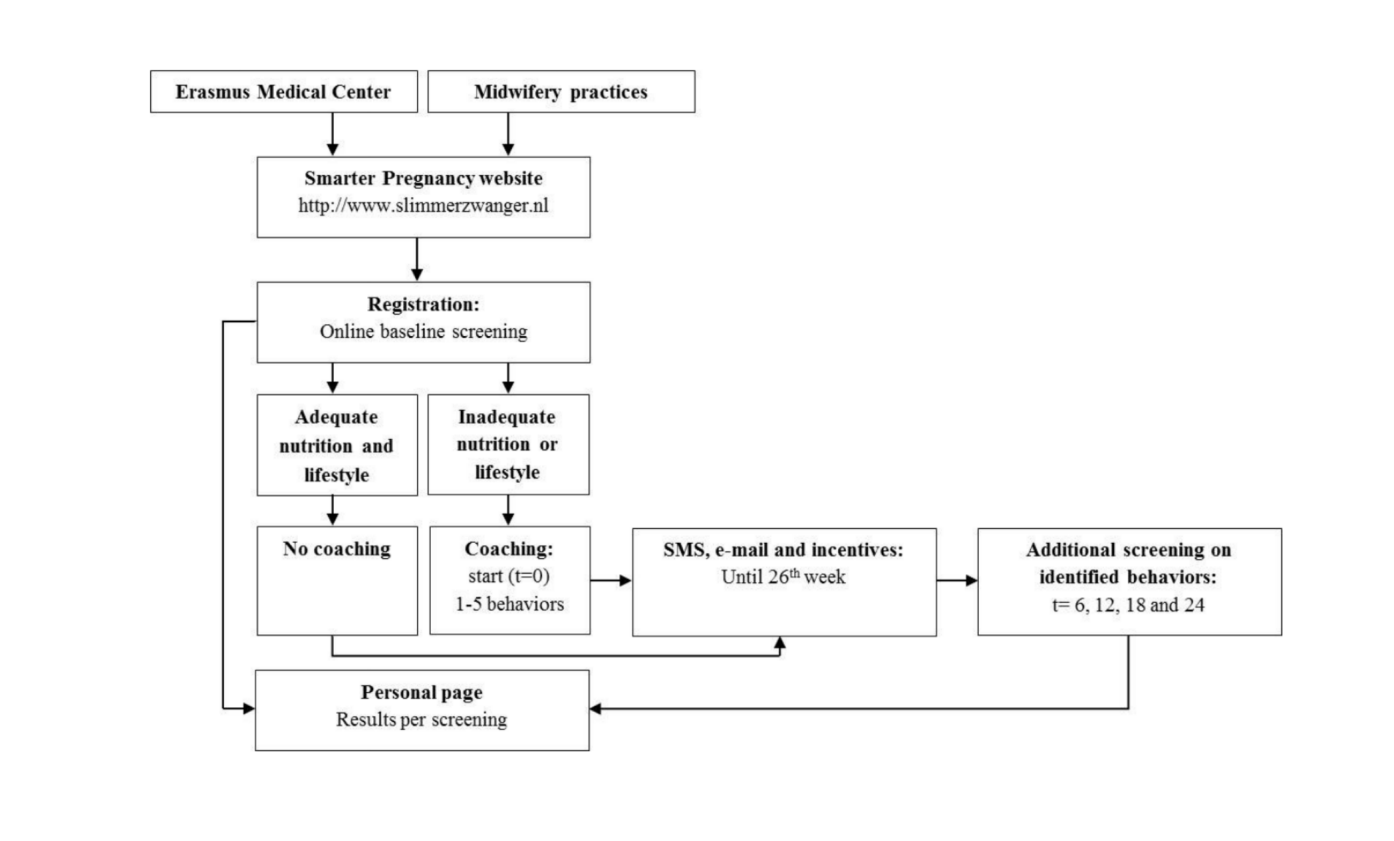
Overview of the Web-based Smarter Pregnancy program: registration, identification of inadequate nutrition and lifestyle behaviors, and coaching. SMS: short message service.

The content of the individual coaching consisted of the baseline screening and follow-up screening at 6, 12, 18, and 24 weeks of the program. Coaching also included a maximum of three interventions per week comprised of short message service (SMS) text messaging and email messages containing tips, recommendations, vouchers, seasonal recipes, and additional questions addressing behavior, pregnancy status, body mass index (BMI), and adequacy of the diet. Every 6 weeks, participants were invited to complete a short, online, follow-up screening survey to monitor the change in their inadequate nutrition and lifestyle behaviors. Results from the screening session compared to the previous screening sessions were shown on their personal page (see Figure 1). This page also provided access to additional modules (ie, applications) to support physical activity, an agenda to improve the compliance of hospital appointments and intake of medication, and a module to monitor the safety of prescribed medication. A summary of all individual results were available to be obtained at any point by the participant, and to be handed over or sent by email to the health care professional for further evaluation and support of preconceptional and antenatal care.

This mHealth platform complied with the highest rules of legislation for medical devices in Europe; therefore, it received the Conformité Européenne, classe 1 (CE-1), classification (2013) and can be used to improve the quality of medical care.

### Statistical Analysis

We analyzed all participants who completed or prematurely resigned from the platform. Compliance was defined by the percentage of participants who completed the 6-month program. Usability was assessed using a digital evaluation form containing 26 questions whose answers were scored using a 4-point Likert scale; the ratings were *negative*, *neutral*, *positive*, and *very positive*. This was used to report on participants' satisfaction with the platform, which was subdivided into three categories: (1) design and interface, (2) content and coaching, and (3) perception and personal benefit. General characteristics and lifestyle behaviors were compared using chi-square tests for proportions, and *t* tests and Mann Whitney U tests for continuous variables.

Using a generalized estimating equation (GEE) model with an independent working correlation matrix, we modeled the fraction that scored *adequate* at each of the follow-up time points. In order to minimize selection bias, we used multiple imputation models to handle missing data of the participants who prematurely resigned. Therefore, a separate model was built for each of the five lifestyle behaviors of interest using all available information on each of the time points, as well as the subgroup indicators to impute the missing values. For each nutrition and lifestyle behavior, we examined those individuals that scored *inadequate* at baseline.

Subgroup analyses were performed between (1) normal weight and overweight or obese women defined as having a BMI of <25.0 and ≥25.0 kg/m^2^, respectively, (2) nonpregnant and pregnant women at the start of the program, and (3) women-only participants and couples, who were defined as the woman and her male partner who followed his own personal coaching program at the same time, which was also dependent on pregnancy status. To create the area under the curve (AUC) of the linear predictor as an overall measure of effectiveness of the program, we calculated the average of the log odds ratio at the specific time points. For each subgroup, this average was compared with that of its complement (eg, obese versus nonobese, pregnant versus nonpregnant, and couples versus women without a participating male partner). SPSS version 21.0 (IBM Corp, Armonk, NY) software package was used and the level of significance was set to .05 for all analyses.

### Ethical Approval

All data were anonymously processed. This survey was conducted according to the guidelines laid down in the Declaration of Helsinki and all procedures involving patients were approved by the Medical Ethical and Institutional Review Board of the Erasmus MC, University Medical Center, Rotterdam, the Netherlands. Digital informed consent was obtained from all participants, allowing us to use the data for analysis.

## Results

### Compliance and Usability

**Figure 2:**
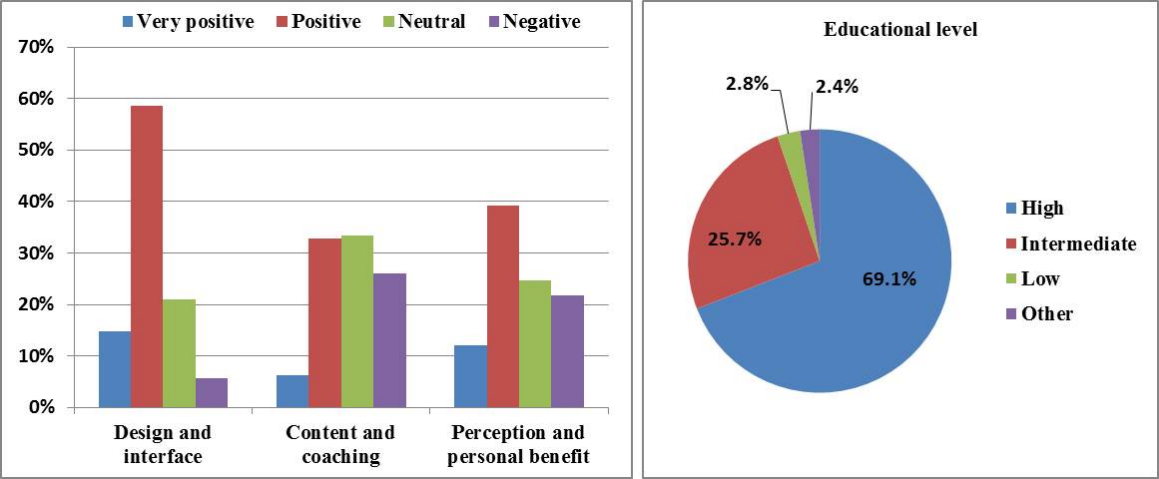
Results of the evaluation of usability based on 357 evaluation forms. Usability of the Smarter Pregnancy program was subdivided into three program characteristics (left) and by participant educational levels (right).

Study compliance was 64.86% (1218/1878) among all participants who activated the program. Additional digital evaluation forms sent every 4 months to new participating women were received from 357 women out of 1878 (19.01%), of which 69.2% (247/357) were highly educated. The usability of the program was judged as *positive* or *very positive* by 54.7% of participants, and ranged from 39.2% (content and coaching) to 73.4% (design and interface) (see Figure 2).

### Baseline Characteristics

**Figure 3:**
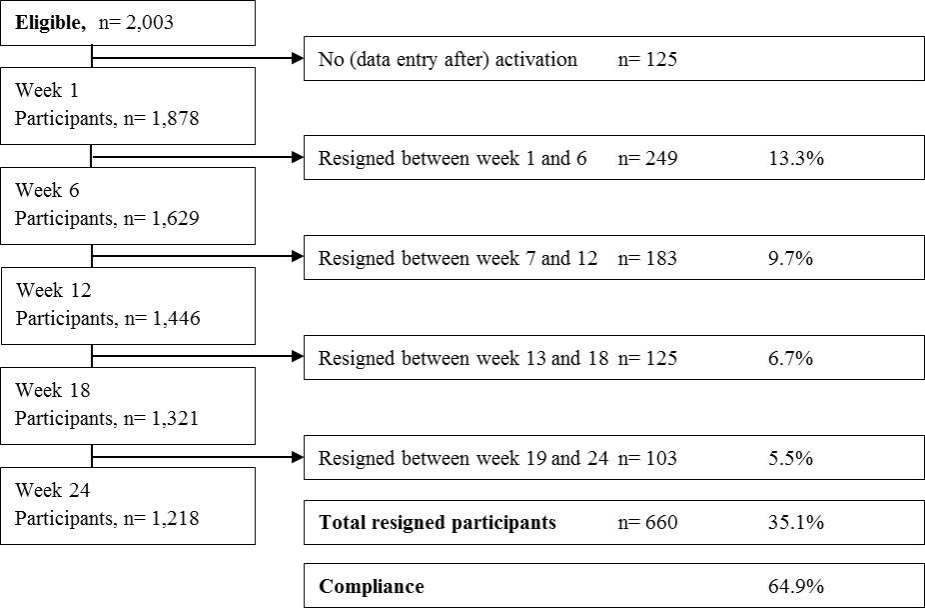
Flowchart of the Smarter Pregnancy survey. Percentages are based on total participants (n=1878) in week 1.

We evaluated 1878 out of 2003 (93.76%) participants after exclusion of 125 (6.24%); these participants were excluded because of nonactivation due to incomplete registration or no data entry after subscribing to the application (see Figure 3). The baseline characteristics of the cohort (n=1878) who completed or prematurely resigned from the platform are depicted in Table 1. They are classified according to gender and further subdivided into groups that (1) completed the last screening and (2) resigned prematurely from the platform. No significant differences were observed in women and men that completed or resigned prematurely from the platform with regard to age, height, BMI, percentage of overweight and obesity, mean vegetable and fruit intake, percentage of inadequate folic acid supplement, and tobacco and alcohol use. The woman-to-man ratio of the participants was 4.3 to 1. Of the total group of 1525 registered women, 603 (39.54%) reported to be pregnant at baseline, of which 416 (69.0%) completed the program and 187 (31.0%) prematurely resigned (*P* =.04).

**Table 1:** Baseline characteristics of participants.

Baseline characteristics	Women (n=1525)			Men (n=353)		
	Completed (n=1003)	Stopped (n=522)	*P*	Completed (n=215)	Stopped (n=138)	*P*
**General**						
Age (years), median (IQR^a^)	31.2 (27.7-34.6)	31.5 (27.9-35.2)	.81^b^	33.7 (30.1-37.0)	34.6 (30.4-38.1)	.64^b^
Height (cm), median (IQR)	169.0 (164.0-174.0)	170.0 (165.0-175.0)	.53^b^	183.0 (179.0-190.0)	185.0 (181.0-188.0)	.16^b^
Pregnant (yes), n (%)	416 (41.48)	187 (35.9)	.04^c^	N/A^d^	N/A	N/A
**Body mass index (BMI) (kg/m^2^)**						
Total group BMI, median (IQR)	24.0 (21.3-27.6)	24.0 (21.7-27.0)	.53^b^	25.2 (23.7-27.8)	25.3 (23.2-27.5)	.30^b^
Overweight (BMI 25-29.99), median (IQR)	27.1 (25.8-28.4)	26.7 (25.9-28.1)	.25^b^	26.6 (25.5-28.1)	27.2 (25.9-28.2)	.48^b^
Overweight, n (%)	266 (26.52)	139 (26.7)		96 (44.7)	62 (45.0)	
Obese (BMI 30-60), median (IQR)	32.9 (31.3-35.8)	32.7 (31.2-36.1)	.52^b^	31.3 (30.8-35.1)	31.7 (30.3-35.1)	.42^b^
Obese, n (%)	141 (14.06)	68 (13.0)		22 (10.2)	10 (7.2)	
**Nutrition**						
Total group vegetable intake (g/day), median (IQR)	135.7 (96.4-185.7)	142.9 (100.0-185.7)	.90^b^	142.9 (100.0-192.9)	150.0 (107.1-185.7)	.88^b^
Inadequate vegetable intake (<200 g/day), n(%)	785 (78.27)	416 (79.9)	.23^c^	162 (75.3)	110 (79.7)	.19^c^
Total group fruit intake (pieces/day), median (IQR)	2.3 (1.3-3.4)	2.1 (1.3-3.3)	.32^e^	1.4 (0.7-2.3)	1.4 (0.5-2.2)	.46^e^
Inadequate fruit intake (<2 pieces/day), n (%)	427 (42.57)	232 (44.6)	.23^c^	139 (64.7)	92 (66.7)	.29^c^
**Lifestyle**						
Folic acid (no), n (%)	150 (14.96)	72 (13.8)	.59^c^	N/A	N/A	N/A
Smoking (yes), n (%)	119 (11.86)	54 (10.3)	.40^c^	48 (22.3)	27 (19.6)	.60^c^
Alcohol (yes), n (%)	258 (25.72)	165 (31.7)	.02^c^	151 (70.2)	94 (68.1)	.72^c^

^a^IQR: interquartile range.

^b^Independent *t* test.

^c^Pearson chi-square test.

^d^N/A: not applicable.

^e^Mann Whitney U test.

### Baseline Nutrition and Lifestyle Behaviors

Adequate nutrition and lifestyle behaviors at baseline were 21.57% (405/1878) for vegetable intake, 52.61% (988/1878) for fruit intake, 85.44% (1303/1525) for folic acid use, 86.79% (1630/1878) for no tobacco use, and 64.43% (1210/1878) for no alcohol consumption. The most prevalent inadequate behavior among both women and men was vegetable intake, which was 78.75% (1201/1525) and 77.1% (272/353), respectively. Inadequate fruit intake was observed in 43.21% (659/1525) of the women and 65.4% (231/353) of the men, whereas only 14.56% (222/1525) of the women reported no folic acid supplement use. Tobacco use was reported for 11.34% (173/1525) and 21.2% (75/353) of the women and men, respectively. Alcohol consumption was reported in 27.73% (423/1525) of all women and 69.4% (245/353) of all men. Women who resigned from the platform prematurely showed a significantly higher percentage of alcohol consumption of 31.6% (165/522) versus 25.72% (258/1003) (*P* =.02).

### Effectiveness

**Figure 4:**
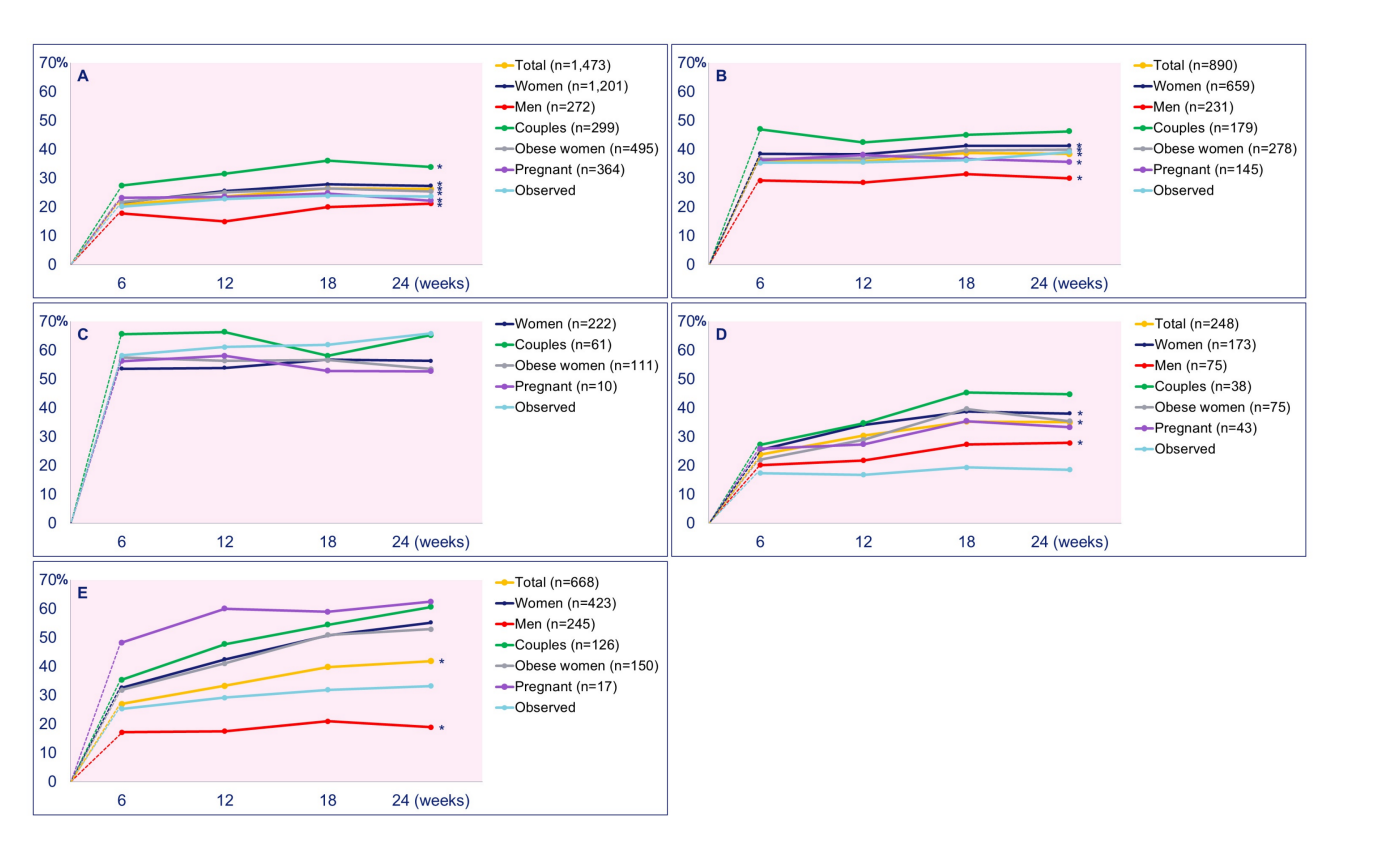
Vegetable intake (A), fruit intake (B), folic acid use (C), tobacco use (D), and alcohol consumption (E) by participants. Improvement of behavior from inadequate at baseline to adequate at every screening point is shown as the percentage (y-axis) of the total group or subgroup. The dotted lines representing the change in relation to baseline are included to improve the interpretation of the graphs. *P<.05 at all screening points. All percentages (per screening point) and areas under the curve, including P values, are included in Multimedia Appendix 1.

Figure 4 depicts the changes in nutrition and lifestyle behaviors of the total and specific subgroups. Results at every follow-up screening point have been compared to baseline values. At baseline, vegetable intake was inadequate in 1473 out of 1878 participants (78.43%). An improvement of 20.9% (95% CI 18.5-23.5) was observed after 6 weeks and persisted to an increase up to 26.3% (95% CI 23.0-29.9) at 6 months (see Figure 4 , A). Inadequate fruit intake was observed in 890 out of 1878 participants (47.39%) at baseline and improved by 36.1% (95% CI 33.0-39.3) and 38.4% (95% CI 34.5-42.5) at 6 weeks and 6 months, respectively (see Figure 4 , B). The figures for inadequate folic acid supplement use observed in 222 out of 1525 women (14.56%) showed a decrease of 53.6% (95% CI 46.8-60.3) and 56.3% (95% CI 48.8-63.6) at 6 weeks and 6 months, respectively (Figure 4 , C). At baseline, the prevalence of tobacco and alcohol use was 248 out of 1878 (13.21%) and 668 out of 1878 (35.57%), respectively. Tobacco and alcohol use were further reduced by 23.8% (95% CI 16.8-32.6) and 27.0% (95% CI 22.4-32.1) at 6 weeks and 35.1% (95% CI 29.1-41.6) and 41.9% (95% CI 35.2-48.9) at 6 months, respectively (Figure 4 , D and E). All percentages are depicted in Multimedia Appendix 1.

### Subgroup: Overweight and Obese Women

Baseline screening revealed 614 out of 1525 (40.26%) and 190 out of 353 (53.8%) overweight and obese women and men, respectively. Subgroup analysis showed patterns of inadequate nutrition and lifestyle behaviors in these women and men comparable to the total group (see Figure 4). The AUCs of the five inadequate lifestyle behaviors were comparable in overweight and obese (BMI ≥25 kg/m^2^) and nonobese (BMI <25 kg/m^2^) women and men (see Multimedia Appendix 1).

### Subgroup: Women Pregnant at Entry

A trend of comparable improvement of vegetable, fruit, and folic acid intake was shown in pregnant and nonpregnant women. Cessation of tobacco and alcohol use was higher in pregnant women although the groups were small (n=10 and n=17, respectively). The AUCs did not differ significantly (see Multimedia Appendix 1).

### Subgroup: Couples

A total of 353 couples were coached, of which 215 (60.9%) completed the 6 months of coaching. The program was most effective on changing inadequate nutrition and lifestyle behaviors, except for tobacco use, when both the women and men used the program compared to the group of women only (see Figure 4).

## Discussion

Smarter Pregnancy is the first CE-1-certified, Web-based, personal mHealth platform tailored to convert inadequate to adequate nutrition and lifestyle behaviors in couples during the prepregnancy and pregnancy periods. This survey highlights the very high prevalence of inadequate intake of vegetables, fruit, and folic acid supplements, as well as tobacco and alcohol use in both women and men in the prepregnancy and pregnancy periods. Previous research by Hammiche et al and Vujkovic et al targeting the same period showed comparable results for inadequate vegetable and fruit intake (32.7-80.6%), inadequate folic acid supplement use (18.9-37.9%), tobacco use (11.3-31.0%), and alcohol use (35.5-66.0%) [6,25]. Screening tools and programs, such as *ZwangerWijzer* [26] and *Healthy Pregnancy 4 All*, have been developed and are being implemented [27,28]. However, routine preconceptional care is still only scarcely available. There is some evidence from other groups substantiating that eHealth and mHealth can support and enhance preventive preconceptional health care interventions.

The strengths of this survey are the high number of participants (n=1878), the high compliance of 64.86% (1218/1878) of participants to complete the 6 months of coaching, the positive feedback of the usability, participation of couples, and the analysis in which selection bias was limited by multiple imputation. The high appreciation of usability and initial effectiveness of this program on improving lifestyle behaviors suggests increased awareness and strong adherence to the given insights and recommendations. A possible explanation for these results is the multifunctional, interactive, and individual character of the coaching, which is distinctive compared to most eHealth and mHealth tools providing information only without taking individual conditions into account. Other strengths are the prospective and automatic data collection, as well as the subgroup analyses addressing the influence of pregnancy status, overweight and obesity, gender, and the participation of individuals or couples.

Our previous research has shown that a short self-administered risk score is a valid method to identify adequate or inadequate vegetable and fruit intake on both food group and nutrient levels [15]. Moreover, the percentages of these inadequate nutrition and lifestyle behaviors are in line with our data from the preconceptional outpatient clinic [6,10]. Limitations of this survey are the absence of validation by biomarkers and, inherent to the design of a survey, the absence of a control group. Moreover, using the Internet and a website in the Dutch language excludes groups using other languages and those having less access to the Internet.

In general, the endless opportunities of mHealth tools and knowing how to access them can be of unprecedented importance, especially with regard to health care. The rise of mobile technology by mobile phones, with more than one billion users worldwide, and other handheld devices also contributes to accessibility regarding online information and recommendations concerning healthy nutrition and lifestyle behaviors during the preconceptional period [29,30]. Couples contemplating pregnancy are often unaware of the availability and importance of these recommendations [5,6,19,31]. Unfortunately, health care professionals are often unfamiliar with up-to-date, evidence-based preconception care; it should be their responsibility to educate and increase patient awareness concerning healthy lifestyle behaviors in order to improve their chances to conceive and ensure a healthy prenatal environment for all couples [5]. Our findings contribute to previous research suggesting that both women and men should be involved in preconceptional care [32]. We demonstrated that the support of the partner by utilizing the same platform increases the effect of this intervention.

It is known that changing inadequate nutrition and lifestyle behaviors and maintaining healthy behavior is hard to accomplish, especially when there is a possibility that the goal to become pregnant will not be reached. Currently, only a small group of women that will not conceive spontaneously and those with a previous complicated pregnancy may receive preconceptional counseling by a health care professional (eg, general practitioner or gynecologist). Because the Smarter Pregnancy program has the potential as an mHealth platform to reach and educate a much larger population, including men, its use and implementation in health care is of interest to patients, health care professionals, and health care insurance companies to reduce health care costs in the future. The initial results of this survey were encouraging; this opens up the opportunity of implementation and conducting randomized controlled trials to further substantiate the findings on changing nutrition and lifestyle behaviors, and to further demonstrate the clinical effectiveness and cost-effectiveness of this mHealth platform in several target groups.

In conclusion, Smarter Pregnancy is a mHealth Web-based coaching platform that has the potential to improve and maintain healthy nutrition and lifestyle behaviors in women as well as men and, in particular, couples in the prepregnancy and pregnancy periods. These findings are important for further improvement of the quality and accessibility of preconceptional and pregnancy care, fertility, pregnancy course and outcome, and ultimately health from the earliest moment and throughout the life course.

## Appendix

Multimedia Appendix 1 Data are presented per risk factor per screening point as the percentage of improvement from inadequate to adequate behavior of the total group or subgroup, including the 95% confidence interval. Area under the curve (AUC) is presented as log odds ratio: AUC subgroup versus AUC complement, difference, and corresponding P value.

## Abbreviations

ASE: attitude, social influence, and self-efficacy
AUC: area under the curve
BMI: body mass index
CE-1: Conformité Européenne, classe 1
GEE: generalized estimating equation
IQR: interquartile range
IVF: in vitro fertilization
MC: Medical Center
mHealth: mobile health
Mrace: Medical Research Advisory Committee
N/A: not applicable
SAG: Stichting Achmea Gezondheidszorg
SMS: short message service
