# Supplementary material for: Impact of an mHealth Platform for Pregnancy on Nutrition and Lifestyle of the Reproductive Population: A Survey
Source: JMIR Mhealth Uhealth. 2016 May 27;4(2):e53. doi: 10.2196/mhealth.5197 (PMC4902855; doi:10.2196/mhealth.5197)
Supplement: Multimedia Appendix 1 [file mhealth_v4i2e53_app1.pdf]

| | N | T=6 | T=12 | T=18 | T=24 | AUC – sub | AUC – compl | Diff | P-value |
|---|---|---|---|---|---|---|---|---|---|
| | | | | | | | | | |
| **Vegetable intake, % (95% CI)** | | | | | | | | | |
| | | | | | | | | | |
| Observed | - | 20.2 | 22.8 | 23.9 | 23.7 | N/A | N/A | N/A | N/A |
| Total | 1,473 | 20.9 (18.5-23.5) | 23.6 (21.2-26.2) | 26.5 (23.0-30.2) | 26.3 (23.0-29.9) | -1.15 | N/A | N/A | N/A |
| Women | 1,201 | 21.6 (19.0-24.4) | 25.6 (22.8-28.6) | 27.9 (24.3-31.9) | 27.4 (23.8-31.4) | -1.07 | -1.56 | 0.49 | .004 |
| Men | 272 | 17.8 (12.8-24.2) | 15.0 (10.2-21.3) | 20.0 (14.5-26.8) | 21.2 (15.5-28.3) | -1.56 | -1.07 | -0.49 | .004 |
| Pregnant | 364 | 23.2 (17.8-29.6) | 23.6 (18.6-29.4) | 24.7 (18.9-31.7) | 22.2 (16.7-29.0) | -1.21 | -1.02 | 0.19 | .177 |
| Overweight and obese | 495 | 21.7 (18.1-25.7) | 25.1 (20.7-30.0) | 26.5 (21.4-32.3) | 25.5 (20.0-31.9) | -1.12 | -1.04 | -0.08 | .519 |
| Couples | 299 | 27.4 (22.4-33.0) | 31.6 (25.5-38.3) | 36.1 (29.5-43.3) | 33.9 (27.8-40.5) | -0.74 | -1.20 | 0.46 | .0009 |
| | | | | | | | | | |
| **Fruit intake, % (95% CI)** | | | | | | | | | |
| | | | | | | | | | |
| Observed | - | 35.4 | 35.6 | 36.2 | 39.1 | N/A | N/A | N/A | N/A |
| Total | 890 | 36.1 (33.0-39.3) | 35.8 (31.9-40.0) | 38.7 (34.0-43.8) | 38.4 (34.5-42.5) | -0.53 | N/A | N/A | N/A |
| Women | 659 | 38.5 (34.6-42.6) | 38.4 (33.1-43.9) | 41.3 (35.4-47.6) | 41.3 (36.1-46.7) | -0.41 | -0.88 | 0.47 | .005 |
| Men | 231 | 29.2 (23.3-35.8) | 28.6 (22.3-35.7) | 31.5 (25.2-38.6) | 30.0 (21.4-40.2) | -0.88 | -0.41 | -0.47 | .005 |
| Pregnant | 145 | 36.3 (28.2-45.2) | 38.1 (30.1-46.8) | 36.7 (26.7-47.9) | 35.7 (24.2-49.0) | -0.54 | -0.38 | 0.17 | .355 |
| Overweight and obese | 278 | 36.7 (29.9-44.0) | 36.8 (28.5-46.0) | 39.6 (30.6-49.3) | 39.9 (30.0-50.7) | -0.48 | -0.37 | 0.11 | .482 |
| Couples | 179 | 47.0 (38.2-56.0) | 42.5 (34.2-51.1) | 45.0 (36.0-54.4) | 46.3 (37.1-55.6) | -0.22 | -0.49 | 0.27 | .087 |
| | | | | | | | | | |
| **Folic acid sup. use, % (95% CI)** | | | | | | | | | |
| | | | | | | | | | |
| Observed | - | 58.2 | 61.1 | 61.9 | 65.8 | N/A | N/A | N/A | N/A |
| Women | 222 | 53.6 (46.8-60.3) | 53.9 (46.5-61.1) | 56.8 (48.7-64.5) | 56.3 (48.8-63.6) | 0.18 | N/A | N/A | N/A |
| Pregnant | 10 | 56.2 (2.3-98.6) | 58.0 (3.0-98.4) | 52.9 (8.9-92.8) | 52.7 (7.6-93.8) | -0.72 | 0.21 | -0.93 | .577 |
| Overweight and obese | 111 | 57.5 (47.1-67.2) | 56.4 (46.9-65.5) | 56.6 (45.8-66.8) | 53.5 (42.8-64.0) | 0.27 | 0.09 | 0.18 | .47 |
| Couples | 61 | 65.6 (52.0-77.0) | 66.3 (50.1-79.5) | 58.1 (43.9-71.0) | 65.3 (52.5-76.2) | 0.55 | 0.04 | 0.51 | .099 |
| | | | | | | | | | |
| **Smoking, % (95% CI)** | | | | | | | | | |
| | | | | | | | | | |
| Observed | - | 17.4 | 16.8 | 19.3 | 18.6 | N/A | N/A | N/A | N/A |
| Total | 248 | 23.8 (16.8-32.6) | 30.4 (24.4-37.2) | 35.3 (28.5-42.8) | 35.1 (29.1-41.6) | -0.85 | N/A | N/A | N/A |
| Women | 173 | 25.4 (17.4-35.3) | 34.1 (26.2-42.9) | 38.7 (30.1-48.0) | 38.1 (29.7-47.4) | -0.72 | -1.18 | 0.46 | .110 |
| Men | 75 | 20.2 (11.3-33.4) | 21.8 (13.5-33.3) | 27.4 (16.7-41.5) | 27.9 (16.9-42.5) | -1.18 | -0.72 | -0.46 | .110 |
| Pregnant | 43 | 25.8 (12.2-46.6) | 27.4 (15.1-44.4) | 35.5 (15.5-62.1) | 33.3 (16.9-55.0) | -0.86 | -0.68 | -0.18 | .617 |

| | | | | | | | | | |
|---|---|---|---|---|---|---|---|---|---|
| Overweight and obese | 75 | 22.0 (12.7-35.5) | 29.0 (18.0-43.1) | 39.7 (25.5-55.8) | 35.4 (22.4-51.0) | -0.84 | -0.65 | -0.19 | .552 |
| Couples | 38 | 27.2 (12.9-48.4) | 34.7 (19.9-53.1) | 45.3 (29.8-61.7) | 44.7 (29.0-61.6) | -0.63 | -0.75 | 0.12 | .736 |
| | | | | | | | | | |
| **Alcohol consumption, % (95% CI)** | | | | | | | | | |
| | | | | | | | | | |
| Observed | - | 25.3 | 29.2 | 31.9 | 33.3 | N/A | N/A | N/A | N/A |
| Total | 668 | 27.0 (22.4-32.1) | 33.3 (29.8-37.1) | 39.8 (34.3-45.6) | 41.9 (35.2-48.9) | 0.63 | N/A | N/A | N/A |
| Women | 423 | 32.7 (27.2-38.6) | 42.5 (37.0-48.0) | 50.7 (44.3-57.1) | 55.2 (46.1-63.9) | -0.22 | -1.49 | 1.27 | .031 |
| Men | 245 | 17.2 (12.3-23.5) | 17.5 (12.7-23.7) | 21.0 (14.6-29.2) | 18.9 (13.7-25.5) | -1.49 | -0.22 | -1.27 | .031 |
| Pregnant | 17 | 48.2 (25.9-71.3) | 60.0 (35.5-80.4) | 58.9 (31.8-81.5) | 62.4 (35.5-83.3) | 0.22 | -0.24 | 0.46 | .325 |
| Overweight and obese | 150 | 31.9 (24.1-40.8) | 41.0 (33.3-49.3) | 50.9 (42.0-59.8) | 52.9 (37.6-67.7) | -0.25 | -0.20 | -0.05 | .788 |
| Couples | 126 | 35.4 (26.4-45.5) | 47.8 (36.0-59.8) | 54.5 (41.1-67.2) | 60.7 (50.1-70.3) | -0.04 | -0.30 | 0.26 | .207 |
